# Clinical measurement of the thoracic kyphosis. A study of the intra-rater reliability in subjects with and without shoulder pain

**DOI:** 10.1186/1471-2474-11-39

**Published:** 2010-03-01

**Authors:** Jeremy S Lewis, Rachel E Valentine

**Affiliations:** 1 Therapy Department, Chelsea and Westminster Hospital NHS Foundation Trust, 369 Fulham Road, London, SW10 9NH, UK; 2 Therapy Department, St George's Hospital, London, UK; 3 Visiting Reader, St George's University of London/Kingston University, London, UK

## Abstract

**Background:**

Clinical sagittal plane assessment of the thoracic kyphosis angle is considered an essential component of the postural examination of patients presenting with upper body pain syndromes. Cervical headaches and conditions involving the shoulder, such as subacromial pain syndrome, have all been associated with an increase in the thoracic kyphosis. Concomitantly a decrease in the thoracic kyphosis as a result of a stretching and strengthening rehabilitation programme is believed to be associated with a reduction in symptoms and pain and improvement in function. Clinicians generally measure the sagittal plane kyphosis angle visually. There is no certainty that this method is reliable or is capable of measuring angular changes over time or in response to intervention. As such a simple and reliable clinical method of measuring the thoracic kyphosis would enable clinicians to record this information. The aim of this investigation was to determine the intra-tester reliability of measuring the thoracic kyphosis angle using a clinical method

**Methods:**

Measurements were made in 45 subjects with and 45 subjects without upper body symptoms. Measurements were made with the subjects in relaxed standing. Two gravity dependent inclinometers were used to measure the kyphosis. The first was placed over the region of the 1^st ^and 2^nd ^thoracic spinous processes. The other, over the region of the 12^th ^thoracic and 1^st ^lumbar spinous processes. The angle produced by each inclinometer was measured 3 times in succession. Each set of 3 measurements was made on two occasions (separated by a minimum of 30 minutes and additional data collection involving 46 further measurements of posture and movement on the same and an additional subject before the thoracic kyphosis measurements were re-measured) by one rater. The reliability of the measurements was analyzed using 2-way ANOVA intraclass correlation coefficients (ICC), 95% confidence intervals (CI) and standard error of measurement (SEM) for precision, for a single measurement [ICC(single)] and the average of 3 measures [ICC(average)]. The assessor remained 'blinded' to data input and the measurements were staggered to reduce examiner bias.

**Results:**

The measurement of the thoracic kyphosis as used in this investigation was found to have excellent intra-rater reliability for both subjects with and without symptoms. The ICC(single) results for the subjects without symptoms were, .95; (95% CI .91-.97). The corresponding ICC(average) results were; .97; (95% CI .95-.99). The results for the subjects with symptoms were; 93; (95% CI .88-.96), for ICC(single) and for ICC(average); .97; (95% CI .94-.98). The SEM results for subjects without and with symptoms were 1.0° and 1.7°, respectively.

**Conclusions:**

The findings of this immediate test-retest reliability study suggest that the clinical measurement of the thoracic kyphosis using gravity dependent inclinometers demonstrates excellent intra-rater reliability. Additional research is required to determine the inter-rater reliability of this method.

**Trial registration:**

National Research Register: N0060148286

## Background

The thoracic kyphosis is the primary curve of the vertebral column and is comprised of 12 vertebrae[1]. The thoracic kyphosis angle increases with age and the increase is greater in females than males[2,3]. This increase may be attributed to an alteration in the intervertebral disc and endplate height, a loss in the anterior vertebral body height, and an imbalance in the supporting anterior and posterior soft tissues and musculature [4-6]. There has also been a suggestion that psychosocial factors such as; despondency, depression, insecurity and anxiety may lead to an increased kyphosis[7]. Biomechanical data suggest that an increase in the thoracic kyphosis may be associated with significantly higher spinal loads and trunk muscle force in upright stance and this might accelerate degenerative process and contribute to dysfunction and pain[6]. In support of this, Scheuermann's disease, a condition associated with increased thoracic kyphosis is associated with more intense back pain, restricted lung function and employment requiring less activity in comparison to a control group matched for gender and age[8].

Findings from a cross sectional study of 536 people over the age of 65 years suggested that an increased kyphosis in females was subjectively associated with poorer health[3]. An increased thoracic kyphosis has also been associated with diminished physical function[9], impairment of respiratory function[8,10], an increase in cervical pain [11-13], headaches[14] and shoulder conditions such as subacromial pain syndrome[15,16]. The rationale for the relationship between the thoracic kyphosis and the development of subacromial pain syndrome is complex. It has been proposed that as the kyphosis increases the scapulae become more protracted and downwardly rotated leading to a potential compression under the acromion and the subacromial tissues included the subacromial bursa and rotator cuff[11,13,15-20]. There is no authoritative definition of what clinically constitutes an excessive kyphosis, and although the evidence for the relationship between the extent of the kyphosis, scapular position and shoulder pain is scant and largely equivocal[21,22] the assessment of the thoracic kyphosis angle and rehabilitation to reduce an excessive kyphosis is considered an integral part of patient management[15,23-25]. This belief is supported by studies that have both demonstrated that (i) an increase in the kyphosis leads to a reduction in shoulder elevation range[26] and (ii) a reduction in the thoracic kyphosis as a result of a rehabilitation programme involving strengthening and stretching exercises leads to an improvement in the range of shoulder elevation[27]. The gold standard method for measuring the thoracic kyphosis is a standing radiograph. Using this method the Cobb, modified Cobb, computer assisted method for deriving radius of thoracic spine curvature, and thoracic vertebral centroid angles may be measured and calculated[6,28].

Within a clinical setting, a radiological investigation of the kyphosis would not generally be indicated and therefore, a reliable, simple and time efficient method of assessing the thoracic kyphosis would be beneficial in a given patient population. Inclinometers have been used in reliability investigations of range of movement and angular postures [29-31] and potentially represent a method of measuring the thoracic kyphosis.

The aim of this study was to investigate the intra-rater reliability of measuring the thoracic kyphosis using a pair of gravity dependent inclinometers in relaxed standing in subjects with and without shoulder symptoms.

## Methods

### Participants

This study formed part of a series of investigations on upper body posture. Subjects with symptoms were recruited through the orthopaedic and physical therapy out-patient departments in the teaching hospital where the study was conducted. Subjects without symptoms were recruited through personal and public advertisements. Permission to conduct this study was granted by the Riverside Research Ethics Committee, London, UK (Reference number: 04/Q0401/42). All subjects signed witnessed informed consent documents and were aware of all their rights including the right to withdraw from the study at any stage of the investigation.

### Inclusion/exclusion criteria

Inclusion criteria for the subjects with symptoms were; unilateral pain and/or restriction of movement arising from the area of the shoulder (C4/C5 dermatome). Inclusion criteria for the subjects without symptoms were; no lumbar, thoracic, cervical or shoulder or upper limb symptoms. Exclusion criteria for both groups were; an inability to fully communicate in English, subjects younger than 18 years of age, cardiac, respiratory, kidney, circulatory problems, systemic disease, diabetes, and, for female subjects pregnancy or suspicion of pregnancy. For subjects without symptoms additional exclusion criteria were; a history of fractures, treatment or surgery to the lumbar, thoracic, cervical spine and upper limbs.

### Procedure

**Figure 1:**
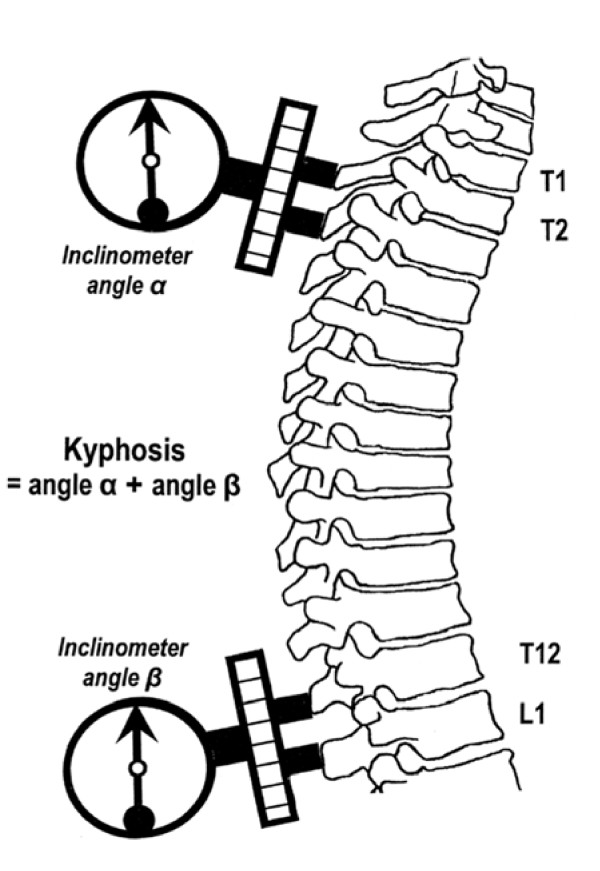
**The thoracic kyphosis angle**. Thoracic kyphosis angle calculated by the summation of the angle recorded by the inclinometer placed over T1 and T2 (angle α) and the angle recorded by the inclinometer placed over T12 and L1 (angle β).

The thoracic kyphosis was measured using two gravity dependent inclinometers (Isomed Inc. 975 SE Sandy Blvd, Portland, OR 97214, USA). As depicted in Figure 1, the feet of the inclinometers were placed over the spinal processes thought to correspond with the 1^st ^and 2^nd ^thoracic spines (T1/2), and, over the 12^th ^thoracic and 1^st ^lumbar spines (T12/L1). These spinal levels were determined by palpation. Measurements were taken in relaxed standing with subjects adopting a natural posture. To achieve this, subjects were requested to swing their arms gently backward and forward 3 times by their sides and stop in a position that felt natural and comfortable to them; to flex and extend their head 3 times gently and stop in a position that felt natural and comfortable to them; and to take 3 breaths and adopt a position that felt natural and comfortable to them. These identical instructions were given to each subject prior to each data collection period. Once this posture had been achieved 6 mm diameter adhesive markers were placed over T1 and T2, and T12 and L1. These levels were identified as follows. The spinous process of the 5^th ^lumbar spine was identified above the sacrum and the L1 and T12 spinous processes were identified and marked by palpating superiorly from this reference point [32]. The 7^th ^cervical vertebra was designated to have the most prominent spinal process[32]. Palpating inferiorly from this reference point the T1 and T2 spinous processes were identified and marked. Once identified subjects were again requested to adopt a posture that felt natural to them and the inclinometers were placed as simultaneously as possible over the markers. Inclinometer measurements were performed 3 times in succession. Following this other postural measurements were taken in supine and standing. The adhesive markers were then removed. This process was termed "postural measurement session 1".

Postural measurement session 1 included a total of 24 separate measurements, involving shoulder range of movement[30], scapular angular and linear measurements[29] and other measurements of posture[33]. Following this postural measurement session another study participant had a set of 24 postural measurements taken. Following the postural measurements made on the second subject the initial subject had a second set of postural measurements including the thoracic kyphosis assessment procedure re-measured. This staggering process (subject 1; 1^st ^postural measurement session, subject 2; 1^st ^postural measurement session, 30 minute break, subject 1; 2^nd ^postural measurement session, subject 2; 2^nd ^postural measurement session, etc) resulted in 46 additional non-related measurements being taken and a 30 minute separation in time before the first and second sets of thoracic kyphosis measurements were completed. The number of measurements made and the process of staggering the patients helped to eliminate examiner bias and the ability to remember or recall the first set of data collection values. This method ensured that by the commencement of the 2^nd ^postural measurement session it was not possible to identify any marks on the skin left by the removal of the adhesive markers prior to their replacement before the second postural measurement session. The investigator verbally relayed the postural measurements to an assistant who transcribed them onto a dedicated assessment page and at no time was the investigator able to see the recorded information.

### Power analysis

This study formed part of a series of studies aiming to investigate measurement reliability as well as relationships of posture for the shoulder and upper body. Walter et al[34] have provided estimates for sample size requirements for reliability studies using intraclass correlation coefficients (ICC). For a true *p*0 of .7 against an alternative *p*1 of .9, based on a 5% significance level and a power of 80% (β = .20) for two raters, or two time points, 19 subjects are required[34,35]. Forty five subjects were recruited into each group (90 in total). This number of subjects was considered adequate to determine the intra-rater reliability for measuring the linear measurement of interest in this study. Forty-six subjects are the required number for a true *p*0 of .8 against an alternative *p*1 of .9[34].

### Statistical analysis

The reliability of the measurements was analysed using intraclass correlation coefficients (ICC), 95% confidence intervals (CI), standard error of measurement (SEM). The descriptive statistics, ICC (Model 2), 95% CI and the SEM statistics were analysed using SPSS version 15 software (SPSS-UK Ltd, St. Andrews House, West Street, Woking, Surrey, GU21 6EB, United Kingdom). The analysis of reliability involved determining the reliability of (i) the first measurement and (ii) the mean of the 3 measurements. Portney and Watkins[36] have described 6 different equations for calculating ICC, and has argued that Model 2 should be used when wishing to confidently generalize the findings of a reliability trial of a particular method of measurement to equally trained clinicians, and Model 3 should be selected when an investigator is interested in establishing the reliability of a measurement procedure for one specific data collection experience without the intention to generalize the findings to equally trained clinicians. ICC (model 2) was used in the current analysis. A 2-way ANOVA ICC was used to compute the reliability and precision (SEM) of a single measurement [ICC (single)] and the average of 3 measurements [ICC (average)]. ICC (single) was analysed by selecting the options; two-way random, single measure, absolute agreement and ICC (average) was analysed by selecting; two-way random, average measure, absolute agreement. The data used in the analysis for the ICC (average) involved taking the mean value of the first set of three measurements and comparing this to the mean value of the second set of three measurements. Portney and Watkins[36] suggested that ICC values above .75 are indicative of good reliability, and those below .75 should be considered as poor to moderate, and in addition, reliability should exceed .90 to ensure reasonable validity.

## Results

Ninety subjects were recruited for this investigation. Of the 45 recruited in the group of subjects with symptoms 23 (51%) females and 22 male subjects (49%). Their combined mean age was 43 years (range 19-84 years), mean height, 1.7 m (range 1.5-1.9 m), and mean weight, 71.4 kg (range 49-90 kg). For the group without symptoms (n = 45) there were 24 (53%) female subjects and 21 male subjects (47%). Their combined mean age was 32 years (range 23-56 years), means height, 1.7 m (range 1.6-1.9 m), and mean weight, 70.4 kg (range 50-110 kg). Patients were referred from orthopaedic surgeons and general practitioners and the diagnoses written on the referral were recorded. The most common diagnoses for the symptomatic subjects were; non-specific shoulder pain (n = 21), and rotator cuff tendinopathy (n = 12). Other diagnoses included; frozen shoulder (n = 2), acromioclavicular joint pain (n = 2), glenohumeral instability (n = 2), stable humeral fractures (n = 1) and stable scapular fractures (n = 1).

For the subjects without symptoms the ICC(single) results for the T1, T2 measurement and T12/L1 measurement respectively were; .92 (95%CI,89-.96) and .89 (95%CI .81-.94). The ICC(average) results for the same measurements were .96 (95%CI .92-.98) and .94 (95%CI .89-.97). The ICC(single) and ICC(average) for the primary outcome measure of the combined T1/T2 and T12/L1 (kyphosis) measurements respectively were .95 (95%CI .91-.97) and .97 (95%CI .95-.99). The SEM result based on the ICC(average) data for the combined T1/T2 and T12/L1 (kyphosis) measurement was 1.0°.

For the subjects with symptoms the ICC(single) results for the T1/T2 measurement and T12/L1 measurement respectively were; .93 (95%CI .87-.96) and .84 (95%CI .73-.91). The ICC(average) results for the same measurements were .96 (95%CI .93-.98) and .92 (95%CI .84-.95). The ICC(single) and ICC(average) for the primary outcome measure of the combined T1/T2 and T12/L1 (kyphosis) measurements respectively were .93 (95%CI .88-.96) and .97 (95%CI .94-.98). The SEM result based on the ICC(average) data for the combined T1/T2 and T12/L1 (kyphosis) measurement was 1.7°. This information, together with mean, standard deviation and range for both groups, together with the SEM results for the ICC(single and average) are reproduced in tabular form in Table 1.

**Table 1:** Reliability statistics for the subjects with and without symptoms

Measurement	Mean(SD)	Range	ICC-single (95%CI)	SEM from ICC-single	ICC-average (95%CI)	SEM from ICC-average
**Subjects without symptoms**						

**T1/2 and T12/L1**	35.5°(6.0°)	5°-62°	.95 (.91-.97)	2.4°	.97 (.95-.99)	1.0°

**T1/T2**	24.9°(8.1°)	2°-49°	.92 (.89-.96)	2.3°	.96 (.92-.98)	1.6°

**T12/L1**	10.5°(4.1°)	0°-23°	.89 (.81-.94)	1.4°	.94 (.89-.97)	1.0°

**Subjects with symptoms**						

**T1/2 and T12/L1**	37.6°(9.5°)	11°-62°	.93 (.88-.96)	2.5°	.97 (.94-.98)	1.7°

**T1/T2**	26.6°(7.6°)	7°-51°	.93 (.87-.96)	2.0°	.96 (.93-.98)	1.5°

**T12/L1**	11.0°(3.7°)	1°-23°	.84 (.73-.91)	1.5°	.92 (.84-.98)	1.1°

Abbreviations: T1 (1^st ^thoracic spinous process), T2 (2^nd ^thoracic spinous process), T12 (12^th ^thoracic spinous process), L1 (1^st ^lumbar spinous process), ICC (intraclass correlation coefficient), SEM (standard error of measurement)

## Discussion

Extension of the thoracic spine contributes to normal elevation of the shoulder[37,38], and regardless of age, approximately 15° of thoracic extension is required for full bilateral arm elevation[37], with unilateral arm elevation requiring approximately 9° of thoracic extension[38]. Of relevance, a large thoracic kyphosis has been associated with reduced arm elevation in older subjects[37] and a post-rehabilitation reduction in the kyphosis resulted in increased shoulder elevation range[27]. Therefore a simple reliable clinical method to measure the thoracic kyphosis may be useful to determine how the kyphosis changes over time as well as the influence of individual or a collection of techniques aimed at reducing the kyphosis. This may enable enhanced understanding of interventions that reduce the kyphosis and if this reduction correlates with an improvement in function and reduction in shoulder pain. The results of this investigation suggest excellent intra-tester reliability for the clinical measurement of the thoracic kyphosis, as used in this study. Although the differences in the ICC(single) and ICC(average) results were small and by themselves do not support recommendations for measuring the kyphosis on one occasion or by calculating the mean of three successive measurements, the SEM findings suggest that there may be less error associated with the clinical measurement using the inclinometer method when the mean of three measurements is employed. This is because a smaller SEM is associated with a more reliable method. In subjects without symptoms, the SEM for ICC(single) for the kyphosis (combined T1/2 and T12/L1) measurements was 2.4° and the SEM for ICC(average) was 1.0°. For subjects with symptoms the SEM for ICC(single) for the kyphosis (combined T1/2 and T12/L1) measurements was 2.5° and the SEM for ICC(average) was 1.7°. This suggests that if an individual clinician wishes to measure the kyphosis using the mean of three sequential measurements, prior to and following an intervention, such as a stretching, strengthening and mobilisation programme aimed at changing the kyphosis, or in a longitudinal study, investigating the change in kyphosis angle over time, in subjects without symptoms, a decrease in the kyphosis of less than 1.0°, and for subjects with symptoms, a decrease in the kyphosis of less than 1.7°, should be considered as measurement error. Real change may be associated with values greater than these. These values appear to be clinically relevant as Wang et al[27] reported that after a 6 week exercise programme in asymptomatic subjects the upper thoracic curve (C7-T7) became significantly (p < .01) less kyphotic by approximately 3° at rest and during movements of the shoulder. Bullock et al[26] reported a mean increase in the thoracic kyphosis of 17.9° when subjects adopted a slouched posture. The slouched posture was also associated with a significant decrease in shoulder flexion range of movement. The methods to measure the kyphosis used in both of these studies required considerable set up times and equipment that would generally not be possible in normal clinical practice.

Research into the effect of the thoracic kyphosis on shoulder range of movement has frequently employed designs that place the subject into fixed or relatively fixed positions of thoracic kyphosis[26,39]. This potentially would confound an understanding of the role of the kyphosis in shoulder function as this type of comparison produces a relatively unnatural fixed posture. This type of fixed posture would not occur typically in general clinical practice with the exception of conditions such as ankylosing spondylitis or Scheuermann's disease. As such an unconstrained method of reliably measuring the kyphosis would be preferable. The advantage of the method reported in this investigation is that it would allow the patient to adopt their natural posture as part of a cross-sectional or longitudinal assessment of the thoracic kyphosis and not require assessment in extremes of range or in a constrained manner.

The inclinometer used in this investigation is gravity dependent with the weight at the base so that the arrow faces superiorly. A similar method was described by O'Gorman and Jull[40] where the inclinometer used had a downward facing arrow. This requires a different mathematical calculation for the determination of the thoracic kyphosis angle. However, both methods should ultimately produce a comparable finding. O'Gorman and Jull[40] reported that three repeated measures in 20 subjects produced F- values below a critical F-value. The description of methods used suggests that only female subjects without symptoms were included in this reliability study and additionally they did not report ICC, 95%CI or SEM results to more fully determine the reliability of the procedure for measuring the thoracic kyphosis[40]. The method described in the current investigation has been used by others[22,41] but the reliability investigation only included 15 subjects with and without symptoms. Lewis et al [22] reported ICC 2,1 results of .94 (95%CI .83- .98) with an SEM of 2.5°. These results are comparable with the findings of the current study.

A limitation of this study is that there is no certainty that this method is stable over time. It was determined that if the kyphosis was measured on separate days or after one week, then potential natural variations in posture occurring as a result of sporting, vocational or routine activity may artificially confound the reliability results. Another limitation is that there is no certainty that the spinal landmarks T1, T2, T12 and L1 were accurately palpated. Although the method used in this investigation is routinely used clinically in an attempt to identify spinous processes there is uncertainty as to the accuracy of clinical methods to identify bony spinal landmarks [42-44]. It would be possible to determine the validity of the palpation technique using radiographs. This could be the focus of future research. Although this method appears to be very reliable, there is no certainty that the procedure is an accurate or valid measurement of the actually anatomical thoracic kyphosis. Future work should compare this method with the Cobb, modified Cobb, computer assisted method for deriving radius of thoracic spine curvature, and thoracic vertebral centroid angles as described by others[6,28]. In addition, another limitation of this study is that only the intra-rater reliability was investigated. To be able to generalise this technique an investigation of the inter-rater reliability of the procedure is necessary.

## Conclusion

In this immediate test-re-test reliability study excellent intra-rater reliability was established using the method described for measuring the thoracic kyphosis angle in subjects with shoulder symptoms as well as subjects without symptoms using a pair of gravity dependent inclinometers. The SEM results provide guidance as to what changes in the kyphosis angle constitute real change as a consequence of intervention or a longitudinal study of the variation in the thoracic kyphosis over time. Future work needs to determine if this method is valid by comparable to direct measurements of the thoracic kyphosis obtained following radiological investigations. In addition to be able to generalise this technique an investigation of the inter-rater reliability of the procedure is required.

## Competing interests

The authors declare that they have no competing interests.

## Authors' contributions

Both authors contributed to the design, data collection and analysis of this investigation. Both authors have read and approved the final manuscript.

## Pre-publication history

The pre-publication history for this paper can be accessed here:

http://www.biomedcentral.com/1471-2474/11/39/prepub
